# Identification and validation of the PARP inhibitor-related gene KANK3 for predicting prognosis and immunotherapeutic response in prostate cancer

**DOI:** 10.7150/jca.113546

**Published:** 2025-09-03

**Authors:** Yan Zhao, Qinghua Wang, Xin Qin, Wei Jiang, Haopeng Li, Mingming Xu, Xilei Li, Hanchu Ye, Juan Zhou, Xi Chen, Gang Wu

**Affiliations:** 1 Department of Urology, Tongji Hospital, School of Medicine, Tongji University, Shanghai 200092, China.; 2 ICU, Tongji Hospital, School of Medicine, Tongji University, Shanghai 200092, China.

**Keywords:** PARP Inhibitor-Related Genes, KANK3, Prognosis, Prostate cancer, Machine Learning, Nomogram.

## Abstract

**Background:** Prostate cancer (PCa), a prevalent malignant neoplasm in men, has its biochemical recurrence-free survival (BCRFS) serving as a critical determinant for patient prognosis. PARP inhibitors have demonstrated potential therapeutic value in the management of PCa. Nevertheless, the precise influence exerted by their associated genes on BCRFS remains elusive.

**Methods:** We selected the differentially expressed genes after treatment with olaparib and defined them as PARP inhibitor-related genes (PIRGs). Consensus clustering was employed to evaluate the relationships among different PIRGs clusters, prognosis, and the immune microenvironment. Univariate COX regression analysis was used to screen the prognosis-related PIRGs, which were then incorporated into multiple machine learning frameworks. The random forest algorithm with the highest C-index was chosen to construct a BCRFS prediction model. A prognostic nomogram was developed based on the risk score and clinical information, and the predictive performance of the model was assessed.

**Results:** In C4 - 2B and LNCaP cell lines, 230 and 58 genes were differentially expressed, respectively. Consensus clustering results showed distinct survival prognoses and immune - infiltrated microenvironments among different groups. The random forest model had a high average C - index in both the training and validation sets. The prognostic model constructed in this study demonstrated a higher C-index compared to the prognostic models from previous studies. High - risk group patients had a poor immunotherapy response. A nomogram based on risk scores and clinical information accurately predicted PCa patients' BCRFS. Cell experiments revealed that KANK3 was downregulated in PCa and upregulated by olaparib treatment. KANK3 overexpression in PCa cell lines inhibited cell proliferation, migration, and invasion, suggesting its oncogenic role in PCa.

**Conclusion:** Our study has described the correlations between PARP inhibitor-related genes and the immune landscape, recurrence after radical prostatectomy, as well as clinical characteristics. The risk score can improve the existing risk stratification system.

## Introduction

Globally, the incidence of PCa shows significant geographical differences. According to the Globocan 2020 data, PCa is the second most common cancer in men worldwide, with approximately 1.414 million new cases, accounting for 7.3% of all malignant tumors. Its incidence is only second to breast cancer and lung cancer^1^. Androgen deprivation therapy (ADT), chemotherapy, immunotherapy, and radiation therapy are all available treatment options for castrate - resistant prostate cancer (CRPC). Recently, PARP inhibitors have also emerged as supplementary treatment choices for this type of cancer^2^-^4^. Biochemical recurrence (BCR) refers to the situation that after PCa patients receive radical treatments (such as radical prostatectomy, radical radiotherapy, etc.), the level of serum prostate-specific antigen (PSA) rises above a certain critical value, and it is generally considered that biochemical recurrence has occurred^5^. This indicates that there may be residual or recurrent tumor cells, but it does not necessarily mean that clinically detectable metastasis or related symptoms will appear. The existing indicators, including Gleason score and PSA, have limitations in accurately predicting the time of BCR in PCA patients^6^, ^7^. Therefore, exploring new biomarkers has important value and far-reaching significance that cannot be ignored in optimizing the diagnosis and treatment process of PCa patients.

Poly (ADP-ribose) polymerase (PARP) inhibitors represent a groundbreaking class of targeted cancer therapies that have significantly advanced the treatment of various cancers, particularly those with specific genetic mutations^8^, ^9^. PARP enzymes, including PARP1 and PARP2, play a crucial role in the repair of single-strand DNA breaks through the base excision repair pathway^10^. When this repair mechanism is compromised, cells accumulate genetic damage, which can lead to cell death. The therapeutic potential of PARP inhibitors stems from their ability to exploit the concept of synthetic lethality. In cancers with deficiencies in homologous recombination repair pathways—such as those with BRCA1 or BRCA2 mutations—cells are already vulnerable due to their inability to repair double-strand DNA breaks effectively^11^. PARP inhibitors further impair the cell's ability to repair single-strand breaks, leading to the accumulation of DNA damage and ultimately causing selective cancer cell death while sparing normal cells.

PARP inhibitors, including drugs such as olaparib, rucaparib, niraparib, and talazoparib, have shown significant efficacy in treating cancers such as ovarian, breast, prostate, and pancreatic cancers^11^-^13^. These drugs have been particularly effective in patients with BRCA mutations or other HRR deficiencies, making them an essential component of personalized cancer treatment. In addition to their use in monotherapy, PARP inhibitors are also being explored in combination with other therapeutic modalities, including chemotherapy and immunotherapy, to enhance their efficacy and overcome resistance mechanisms^14^, ^15^

Overall, PARP inhibitors represent a significant advancement in precision oncology, offering new hope for patients with genetically defined cancers and contributing to the ongoing evolution of cancer treatment strategies^16^-^18^.

In summary, the objective of this study was to delve into the genomic alterations within PCa cell lines subsequent to the administration of PARB inhibitors, explore the influence of PIRGs on prognosis and the immune microenvironment, and establish a predictive model that can accurately forecast the BCRFS of PCa patients through machine learning approaches.

While previous studies have thoroughly elucidated the molecular mechanisms of PARP inhibitor therapy, systematic evaluation of the prognostic value of PIRGs in independent validation cohorts remains scarce. This study constructs an innovative machine learning framework to promote the translational application of genomic signature markers into clinical risk stratification systems. Based on multi-omics data from prostate cancer cohorts, our developed PIRG prognostic model significantly outperforms existing models in C-index evaluation. A clinically practical nomogram was further constructed, integrating PIRG risk scores with clinical information to achieve accurate prediction of biochemical recurrence-free survival. Functional mechanism studies confirmed that KANK3 acts as a key tumor suppressor gene in prostate cancer, and its expression silencing is closely associated with tumor aggressive phenotypes and PARP inhibitor resistance. This study not only confirms the prognostic value of PIRG signature profiles but also provides a theoretical basis for precision diagnosis and treatment strategies in advanced PCa.

## Materials and Methods

### Public data acquisition

We retrieved the PCa cohorts encompassing BCRFS information from public databases. The TCGA cohort was sourced from the TCGA database (https://portal.gdc.cancer.gov). The DKFZ2018 cohort was procured from the cBioportal website (https://www.cbioportal.org/). The sequencing data of cell lines in both the Olaparib treatment group and the control group (GSE116918), along with the PCa cohorts (GSE70769 and GSE189186) incorporating BCRFS, were retrieved and downloaded from the Gene Expression Omnibus (http://www.ncbi.nlm.nih.gov/geo). Regarding the expression data derived from high-throughput sequencing, we transformed it into the TPM format and conducted a log2 transformation. For the microarray data, we employed the normalizeBetweenArrays function within the limma package to rectify systematic deviations among different samples in the microarray experiments, thereby rendering the gene expression data of various samples comparable^19^. Simultaneously, we also downloaded the survival information of the aforementioned cohorts and excluded patients with a survival time of zero months to ensure the reliability of the analysis. The baseline data of patients included in the datasets of this study are shown in Supplementary Table 1.

### Differential expression analysis

The DESeq2 R package was employed to perform differential expression analysis^20^. Genes fulfilling the criteria of |log2fold change| > 0.5 and p-value < 0.05 were designated as differentially expressed genes (DEGs).

### ssGSEA

Each ssGSEA enrichment score reflects the extent to which genes within a particular gene set are upregulated or downregulated in a given sample^21^. We retrieved the gene sets of 28 immune cells from prior studies. By employing the ssGSEA method, the expression matrix was converted into an enrichment matrix, thus obtaining the immune cell infiltration levels of different samples.

### Survival analysis

Survival analysis was carried out by means of the survival R package. For the gene expression data or risk score data of each patient, we employed the survminer package to compute the optimal cut-off value and stratify the patients. Subsequently, Kaplan-Meier analysis was performed on the stratified patients, and the survival curves were generated.

### Consensus clustering analysis

We employed the ConsensusClusterPlus R package to conduct consensus clustering analysis on patients based on the differentially expressed genes associated with prognosis^22^, which were screened through univariate COX regression analysis. The iteration count was configured at 50 times. Eighty percent of the samples were selected for repetitive sampling procedures. The Euclidean distance calculation method was utilized, and the PAC approach was adopted to identify the optimal number of clusters.

### Fitting and validation of machine learning models

In order to obtain the most favorable prognostic model, an overall integration of ten machine learning algorithms, including CoxBoost, Lasso, Ridge, Enet, StepCox, survival-SVM, GBM, plsRcox, RSF, and SuperPC, was executed. Initially, variable screening was performed using algorithms like StepCox, Lasso, CoxBoost, and RSF that possess variable selection capabilities. The selected variables were then incorporated into algorithms capable of model construction for the purpose of model fitting.

For CoxBoost analysis, the CoxBoost R software package was used, and 10-fold cross-validation was adopted to determine the optimal boosting steps. The glmnet R package was utilized to build Lasso, Ridge, and Enet models, where 10-fold cross-validation was employed to find the regularization parameter lambda. The survival-SVM model was fitted using the survivalsvm R package. The gbm R package was used to build the GBM model, and 10-fold cross-validation was carried out. The plsRcox R package was used for fitting the plsRcox model. To construct the RSF model, the rfsrc function from the randomForestSRC R package was employed, with the ntree parameter set at 1000. The SuperPC model was built through the superpc R package, and 10-fold cross-validation was applied to fit the most appropriate model.

### Immune infiltration analysis

We computed the ESTIMATE scores, stromal scores, and immune scores of patients by utilizing the ESTIMATE R package^23^. Furthermore, we retrieved the marker gene sets of 28 immune cells from the literature and employed the ssGSEA algorithm to evaluate the infiltration of these 28 immune cells among different patients. In addition, we utilized the MCP counters algorithm to calculate the infiltration levels of 10 immune cells in various patients for verification purposes.

### Immunotherapy prediction and drug sensitivity prediction

Gene expression data were first subjected to normalization procedures. Subsequently, the TIDE score, dysfunction score, and exclusion score for each patient were computed via the TIDE (Tumor Immune Dysfunction and Exclusion) database (http://tide.dfci.harvard.edu/)^24^.

By leveraging the oncoppredict R package, the standard expression matrix of GDSC2 and the IC50 values of each cell line corresponding to every drug were retrieved from Github (https://github.com/maese005/oncoPredict/tree/main/vignettes). These data were then utilized as the training set to estimate the IC50 values of various drugs for patients within distinct risk groups.

### Construction of nomogram and validation of diagnostic efficacy

Regarding the construction of the prognostic nomogram, clinical data from the TCGA prostate cancer cohort were utilized, including age, clinical T stage (cT), pathological T stage (pT), pathological N stage (pN), calculated Risk_Score, and risk group stratification. Univariate COX regression analysis was first performed on risk scores and clinical parameters to screen for prognosis-related variables. Subsequently, stepwise regression was applied, with the Akaike Information Criterion (AIC) used to quantify model complexity and goodness-of-fit, aiming to identify the optimal model that balances data explanation with minimal free parameters. This process identified Risk_Score and cT as robust independent prognostic factors, forming the final model with a reduced AIC value. Serving as the foundation for nomogram construction using the rms package in R, this model enables prediction of 1-year, 3-year, and 5-year survival probabilities. For the final multivariate COX regression model, the rms package was employed to construct the nomogram, with calibration curves used to assess discrepancies between predicted and observed survival outcomes. Time-dependent AUC curves were generated using the pecr package to evaluate predictive accuracy at distinct time points. To assess clinical utility in decision-making, net benefit analyses of different strategies within a specified threshold range were conducted using the ggDCA package, evaluating the model's practical application value^25^.

### Cell culture and transfection

The PCa cell lines C4-2B and 22RV1 were procured from the Cell Bank of the Chinese Academy of Sciences. These cell lines were cultured in RPMI 1640 medium supplemented with 10% fetal bovine serum within an incubator maintained at 37°C and 5% CO₂. The KANK3 transfection plasmid utilized in our study was purchased from Hunan Youze Biotechnology Company (China), and Lipofectamine 2000 (Invitrogen, USA) was utilized to transfect the plasmid into the cell lines, thereby achieving the overexpression of KANK3.

### Real-time qPCR

Total RNA was isolated from the cell lines by employing the TRIzol kit (Invitrogen, USA). Subsequently, the extracted RNA was reverse transcribed into cDNA through the utilization of the PrimeScript RT kit (Takara, Japan). After that, the cDNA was prepared in a 10 µl reaction system using the TAKARA kit. Next, the cDNA prepared earlier, gene-specific primers, ddH₂O, and 2 × Taq Pro Universal SYBR qPCR master mix were combined for qPCR amplification. The relative expression of the target gene was determined using the 2 - ΔΔct method, with GAPDH acting as the internal reference gene.

### Western blot

PCa cells' protein was extracted using RIPA buffer. Prepared protein standards following kit (Beyotime, Shanghai) instructions. Made BCA solution based on sample count and added to samples/standards. After 30 min at RT, measured 562 nm absorbance with microplate reader (Varioskan LUX, US) to determine protein concentration by BCA. Added 5× loading buffer (4:1 to sample) and incubated at 100°C for 15 min.

Loaded samples on 10% SDS - PAGE gel for electrophoresis and transferred to PVDF membrane. Blocked membrane with 5% BSA for 1 h at RT. Incubated overnight at 4°C with KANK3 and GAPDH antibodies. Detected protein expression by enhanced chemiluminescence and analyzed bands with imaging system.

### Cell function experiments

The KANK3 plasmid was introduced into 22RV1 and C4 - 2B cells to investigate its influence on cell migration and invasion. In the wound healing test, a scratch was created on the cell monolayer, and the movement of cells into the scratch was observed over 24 hours. Regarding the Transwell assay, transfected cells were positioned in the upper chamber with serum - free medium, and the lower chamber contained 30% serum medium. After 36 hours, the migrated cells reaching the lower side of the membrane were enumerated. These experiments assist in clarifying the function of KANK3 in cell migration and invasion and offer data backing.

### EDU assay

Transfected cells were plated in 96 - well plates at 1×10⁴ cells/well. After 24 h incubation, EDU solution was diluted to 50 μM in Edu medium and 100 μL added to each well for another 2 h incubation. Then, cells were fixed and 100 μL Click - iT working solution (Uelandy, Suzhou) was added for staining. Finally, DNA was counterstained and images taken by fluorescence microscope.

### Clonogenic assay

800 transfected cells were seeded in a 6 - well plate. After 10 days, cells were washed twice with PBS, fixed with 4% paraformaldehyde, and stained with crystal violet. Then, the number of cell clones was determined.

### Immunofluorescence

Transfected cells were plated in a 6 - well plate at an appropriate density. Then, they were fixed with 4% paraformaldehyde and permeabilized with 0.2% Triton X - 100 at room temperature. After that, cells were incubated with 200 μL phalloidin working solution for 20 minutes and counterstained with DAPI. Finally, immunofluorescence microscopy was used to observe KANK3 protein distribution.

### Immunohistochemistry

Immunohistochemistry was done on two tissue specimens (1 PCa and 1 normal prostate tissue from Tongji Hospital Urology Dept.). Tissues were fixed in 10% formalin, embedded in paraffin, and sectioned at 4 μm. After dewaxing and rehydration, antigen retrieval was carried out with citrate buffer (pH 6.0) in a microwave. Then, sections were blocked with 3% hydrogen peroxide, incubated with primary antibody at 4 °C overnight. After washing, they were incubated with biotinylated secondary antibody and streptavidin - HRP. Color was developed with DAB and sections counterstained with hematoxylin. Finally, slides were dehydrated, mounted, and analyzed under an optical microscope. Each sample was evaluated by two pathologists.

### Statistical analysis

All bioinformatics analyses were performed using R version 4.1.3. The normality of continuous variables was tested using the single-sample KS test. If the data followed a normal distribution, data analysis was conducted using the t-test or one-way ANOVA analysis of variance. The experiments were repeated three times. A significance level of P < 0.05 was considered statistically significant.

## Results

### Analysis of the differential gene expression following olaparib administration

**Figure 1:**
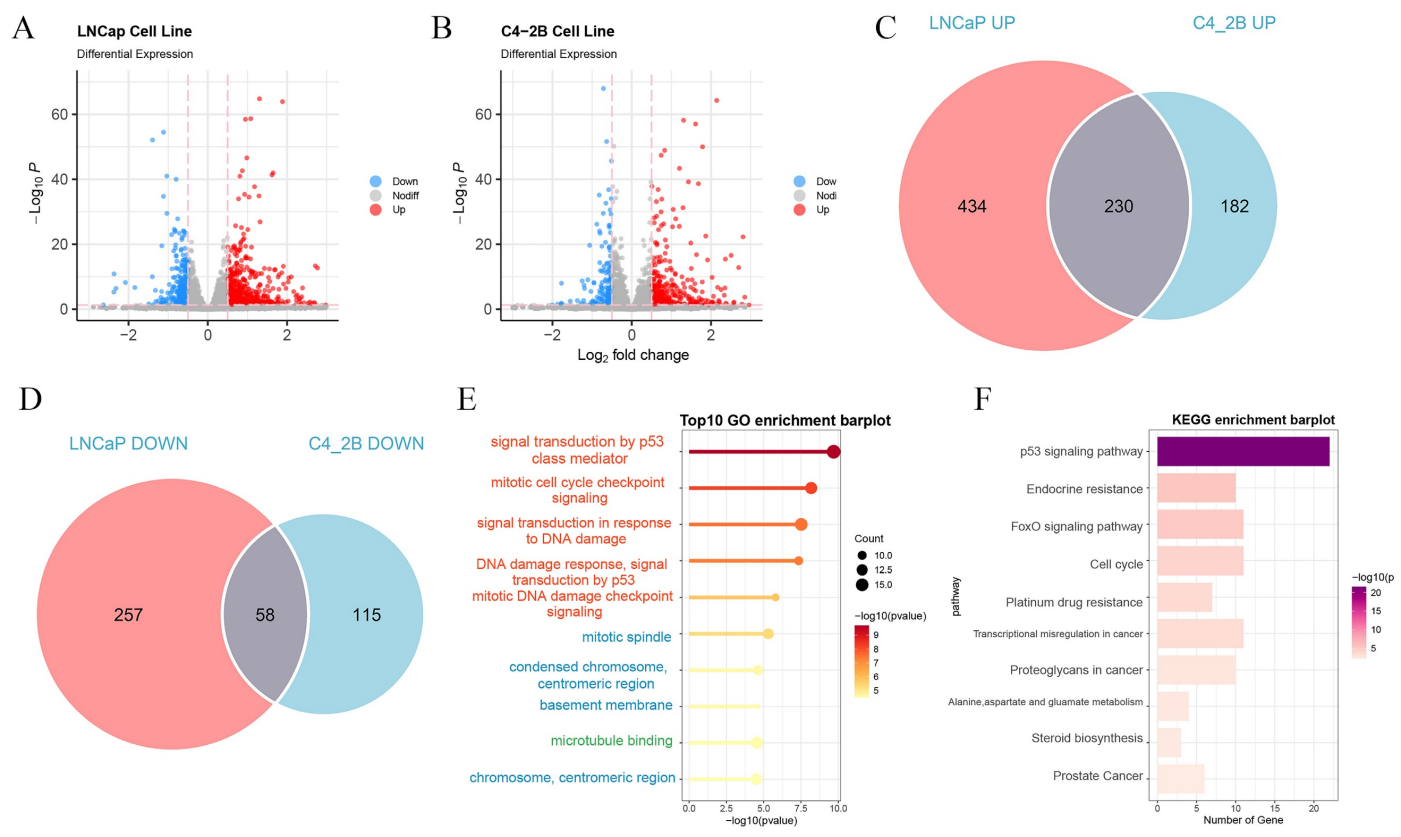
Identify differentially expressed PIRGs in PCa cells. **A**,**B** Volcano plot of genes differentially expressed in LNCaP, C4-2B in dataset GSE189186. **C**,**D** Wayne plot of differentially expressed genes. E Enriched GO terms of the differentially expressed PIRGs. **F** Enriched KEGG pathways of differentially expressed PIRGs.

The GSE189186 dataset was obtained from the GEO database. Subsequently, differential expression analyses were carried out on cell lines prior to and subsequent to Olaparib treatment. Genes fulfilling the criteria of | log2FoldChange | > 0.75 and P < 0.05 were designated as differentially expressed genes. In the LNCaP cell line, a sum of 846 such genes were successfully screened, whereas 430 differentially expressed genes were detected in the C4-2B cell line (Figure 1A - B).

The differentially expressed genes that exhibited concurrent upregulation or downregulation in both cell lines were chosen for further in-depth exploration (Figure 1C - D). These genes were designated as PIRGs, with a total number of 288. The Gene Ontology (GO) enrichment analysis was principally associated with signal transduction by p53 class mediator, mitotic cell cycle checkpoint signaling, signal transduction in response to DNA damage and mitotic DNA damage checkpoint signaling. Notably, the differentially expressed genes were primarily concentrated in KEGG-correlated pathways, including but not limited to the p53 signal pathway, endocrine resistance, FoxO signaling, and cell cycle pathway.

### Unsupervised clustering analysis was carried out on PCa patients with reference to the expression levels of PIRGs

**Figure 2:**
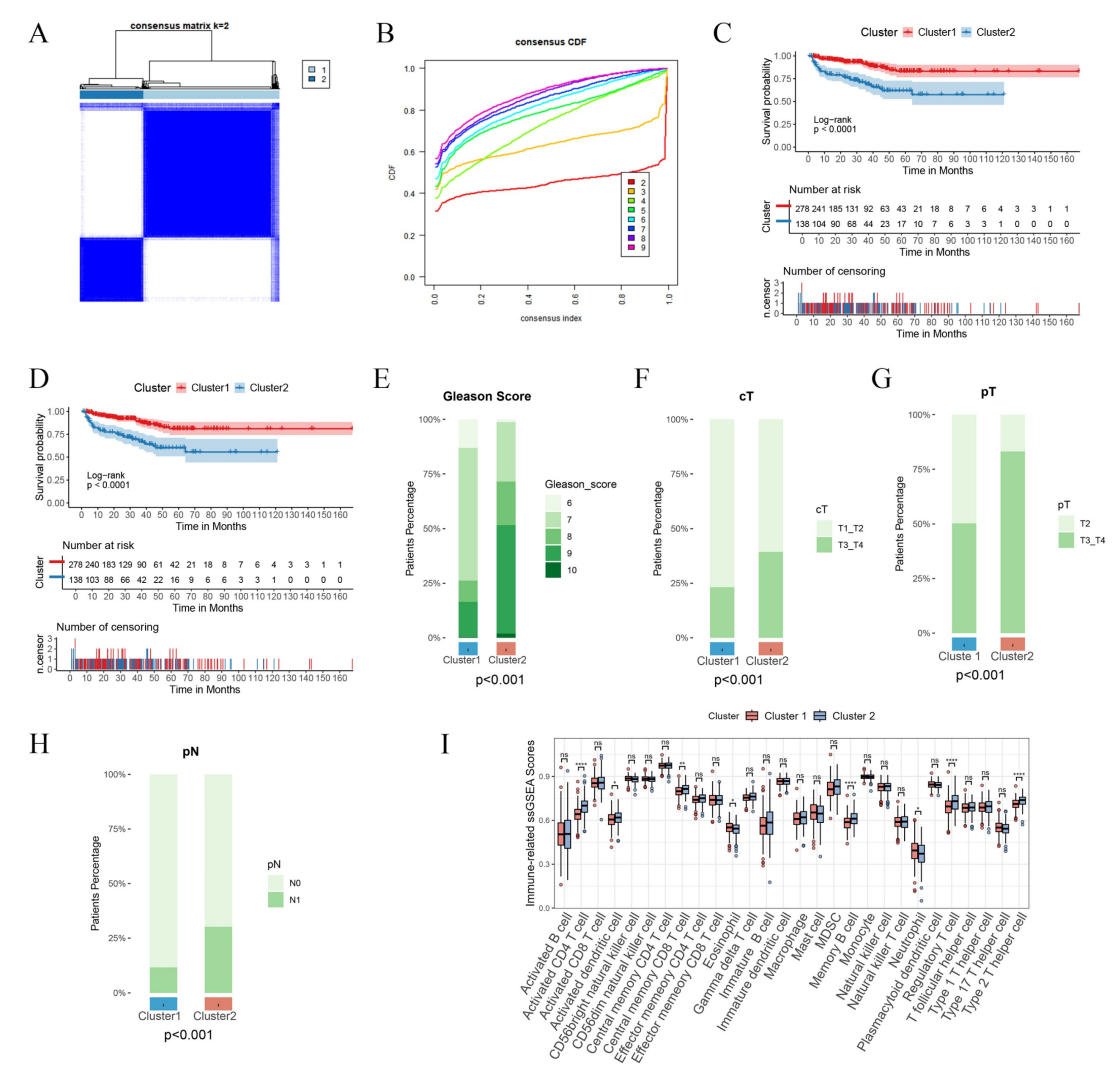
Consensus clustering based on differentially expressed PIRGs: Patients with differentially expressed PIRGs are divided into two clusters by consensus clustering algorithm. **A**,**B** Heat maps and trace curves show consensus clustering of k = 2 groups in sample clustering. When k is 2, the degree of group differentiation is the highest and the group consensus is also the best. **C** Differences in PFS between the two clusters. **D** Differences in BCR between the two clusters. **E**-**H** Differences in clinical information between the two clusters (Gleason score, cT, pT, pN). **I** Difference in immune cell infiltration between the two clusters.

Leveraging the expression profiles of PIRGs in PCa patients, we implemented unsupervised clustering by means of the ConsensusClusterPlus R package. The CDF curve demonstrated a tendency to level off at k = 2, accompanied by a distinct blocky structure, suggesting a notably high stability of sample partitioning at this value of k. Consequently, we elected to stratify the patients into two cohorts (Figure 2A - B).

Subsequently, we delved deeper into the disparities in prognostic outcomes across different patient clusters. It was ascertained that patients belonging to Cluster 1 boasted a superior BCRFS in comparison to those in Cluster 2, with a parallel trend being discernible for PFS as well (Figure 2C - D). Notably, patients in Cluster 1 were characterized by lower Gleason scores and earlier clinical and pathological staging (Figure 2E - H). Moreover, the infiltration of immune cells within the tumors of patients in the two clusters also manifested differences. In contrast to Cluster 1, patients in Cluster 2 displayed augmented infiltration of activated CD4 T cells, central memory CD8 T cells, and memory B cells (Figure 2I). To sum up, disparate expression patterns of PIRGs are potentially correlated with both the prognosis of PCa and the tumor immune microenvironment.

### Construction of prognostic prediction models for biochemical recurrence-free survival (BCRFS) of prostate cancer (PCa) patients employing multiple machine learning methods

**Figure 3:**
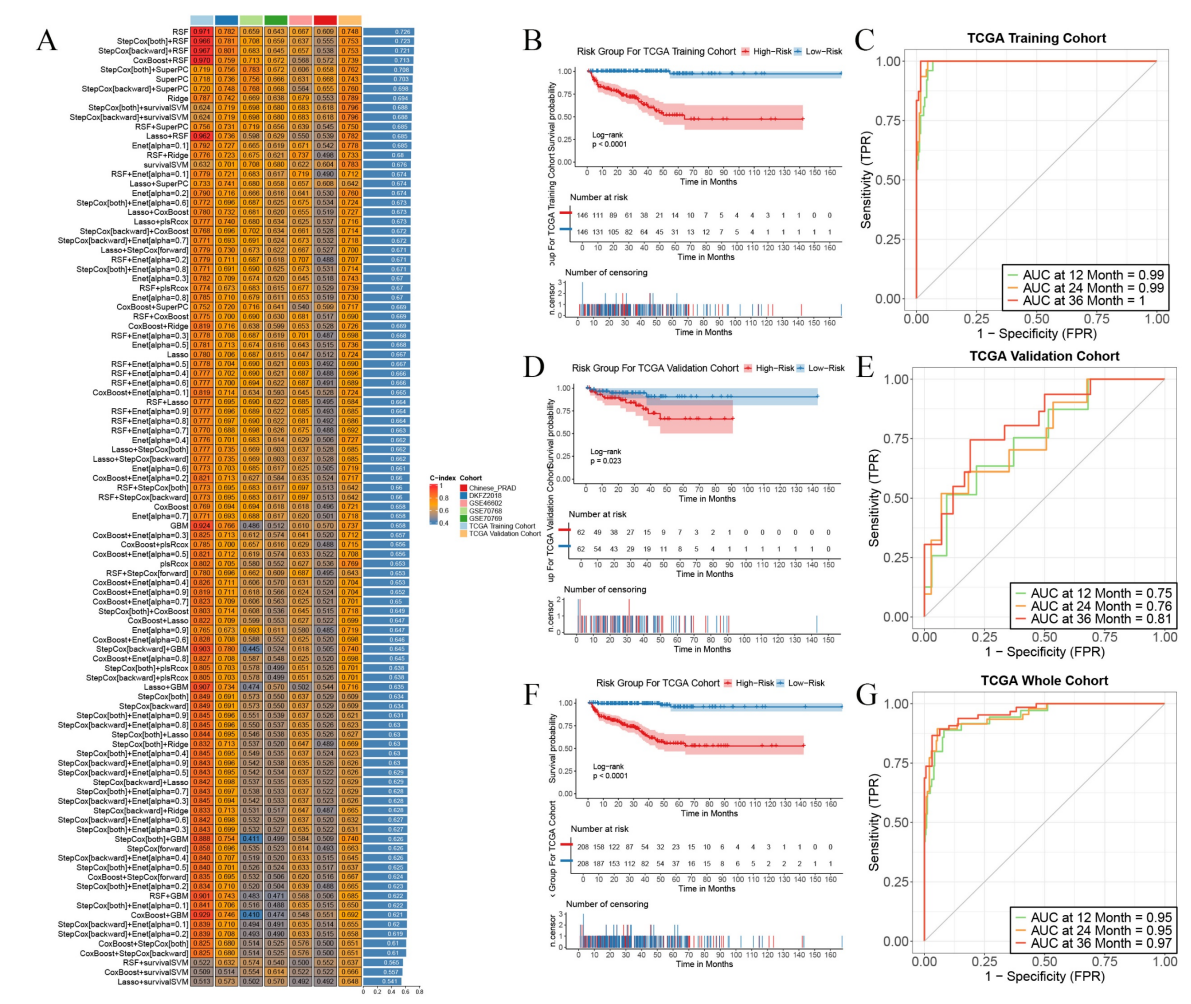
Construction and validation PIRGs prognostic models based on machine learning. **A**. C-index were calculated for each model on all validation datasets. **B**,**D**,**F** TCGA- PCa training set, internal validation set, patient risk score and overall survival status distribution in the overall set. **C**,**E**,**G** ROC curves (reflecting specificity and sensitivity) of PIRGS in predicting 1-, 3-, and 5-year OS in the TCGA Training Cohort, TCGA Validation Cohort, and TCGA Cohort.

Independent validations were subsequently effected in multiple external validation cohorts, with the C-index adopted as the evaluative index to gauge the model's predictive efficacy. The outcomes manifested that the Random Survival Forest (RSF) model boasted the highest average C-index, registering at 0.726. Consequently, we opted for this model for subsequent exploration (Figure 3A). This model was composed of five genes, namely RGS11, MMP24, ASRGL1, KANK3, and BTG2. Relying on the expression magnitudes of these five genes, the RSF model was harnessed to compute the risk scores for each patient in the training set and multiple external validation sets.

We stratified all patients into high-risk and low-risk cohorts in accordance with the median risk score and contrasted the survival endpoints of patients in different risk cohorts in each cohort. We discerned that in the TCGA training cohort (Figure 3B), the TCGA validation cohort (Figure 3C), and the TCGA cohort (Figure 3F), the overall BCRFS of high-risk patients was conspicuously lower than that of low-risk patients (p < 0.05, log-rank test). Receiver Operating Characteristic (ROC) curve analysis divulged that in the TCGA training cohort, the areas under the curve (AUC) for predicting 1-year, 2-year, and 3-year survival attained 0.99, 0.99, and 1, respectively (Figure 3C). In the TCGA validation cohort, they were 0.75, 0.76, and 0.81, respectively (Figure 3E). In the TCGA cohort, they were 0.95, 0.95, and 0.97, respectively (Figure 3G). Additionally, this model also exhibited outstanding predictive competencies in multiple external validation sets, attesting to the robustness of the prognostic model we constructed and its favorable prospects for broad dissemination and application. These results imply that the prediction model erected on the foundation of PIRGs harbors potent prognostic prediction potential in PCa and may function as a novel predictor to steer clinicians' treatment decisions.

**Figure 4:**
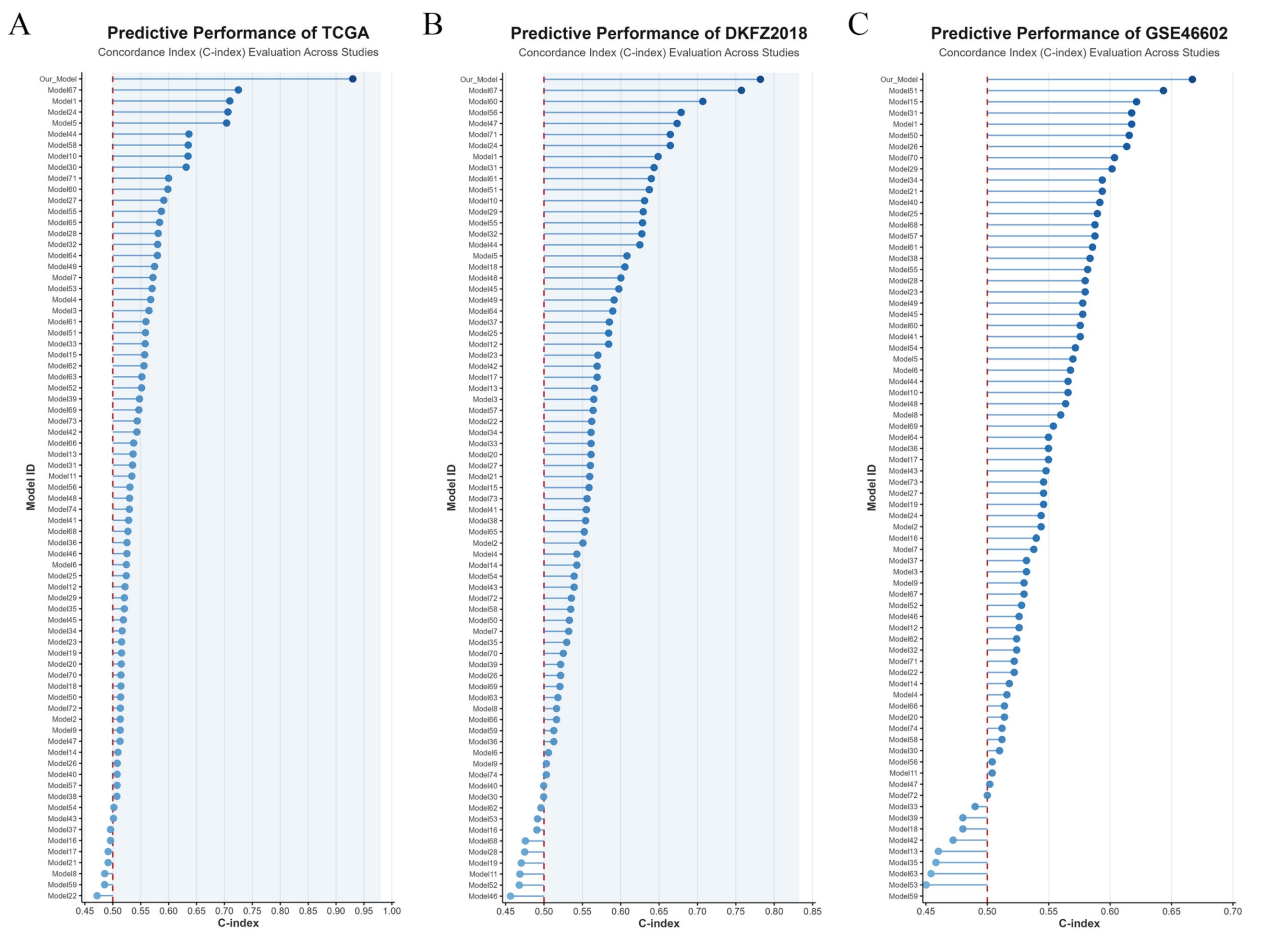
Comparison of PIRGs prognostic models with prognostic models from previous studies. **A**. Comparison of prognostic models based on the TCGA dataset. **B**. Comparison of prognostic models based on the DKFZ2018 cohort. **C**. Comparison of prognostic models based on the GSE46602 dataset.

To further compare the predictive performance of the PIRG-based prognostic model with previously developed BCRFS prediction models for PCa, this study compiled predictive models from dozens of prior investigations and visualized their C-index values across the TCGA, DKFZ2018, and GSE46602 datasets. Notably, the PIRG-derived model demonstrated superior diagnostic efficacy compared to all other validated models, underscoring its enhanced accuracy in predicting biochemical recurrence-free survival (Figure 4A-C).

### Research on the correlation among the tumor immune microenvironment, tumor stemness characteristics, and risk scores

Based on previous studies, PARP inhibitors play crucial roles in enhancing immunogenic cell death, regulating the tumor microenvironment, reversing immunosuppression, synergizing with immunotherapy, and influencing DNA damage repair and immune responses. Therefore, we sought to analyze the relationship between the risk scores of the prognostic model constructed based on PIRGs in PCa and the immune microenvironment of patients.

**Figure 5:**
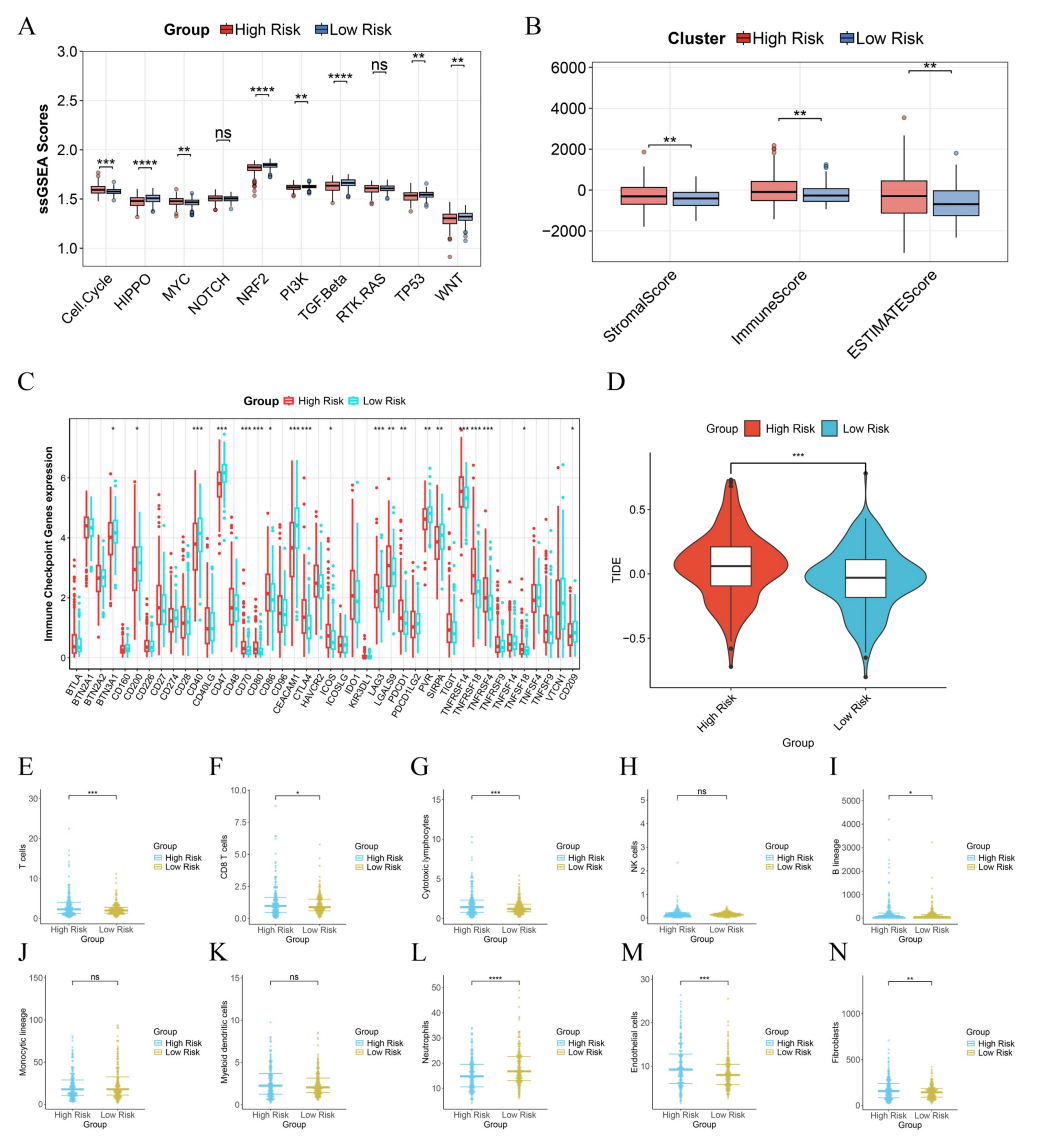
Immune Landscape Associated with PIRGS in PCa. **A**. ssGSEA analysis of the significantly different cancer-related pathways. **B** The Stromal score, the immune score, and the ESTIMATE score were applied to quantify the different immune statuses between the high- and low-risk groups. **C** Single - sample gene set enrichment analysis (ssGSEA) was used to analyze the expression of immune checkpoint genes in high - risk and low - risk groups. **D** The TIDE scores of the high-risk group and the low-risk group. **E-N** Box plots illustrate the infiltration status of T cells, CD8 T cells, cytotoxic lymphocytes, NK cells, B lineage, monocytic lineage, myeloid dendritic cells, neutrophils, endothelial cells, and fibroblasts in the high-risk and low-risk groups.

We calculated the enrichment scores of tumor pathways using the ssGSEA based on the gene sets of oncogenic signaling pathways reported by Sanchez-Vega et al. We found that the high-risk group had higher scores in oncogenic signaling pathways such as the cell cycle, MYC, NOTCH, and RTK, while the low-risk group exhibited relatively higher activity in pathways including HIPPO, NRF2, PI3K, TGFβ, and TP53. This suggests that patients in different risk groups divided by the model constructed based on PIRGs may be regulated by the activity of these pathways, leading to different prognostic differences (Figure 5A).

To evaluate the immune infiltration status of PCa samples, we applied the ESTIMATE algorithm to calculate the immune scores, stromal scores, and ESTIMATE scores of risk subgroups. The immune scores, stromal scores, and ESTIMATE scores of patients in the high-risk group were significantly higher than those in the low-risk group (Figure 5B). Subsequently, we compared the expression levels of 44 major immune checkpoint targets in high-risk and low-risk patients in the TCGA dataset. The results showed that the expression of most immune checkpoint genes in high-risk patients was lower than that in low-risk patients, indicating that risk scores can assist clinicians in better selecting treatment strategies for immune checkpoint inhibitors (Figure 5C).

To assess the effectiveness of immunotherapy in patients of different risk groups, we calculated the TIDE scores of each patient through the TIDE database. The results indicated that the TIDE scores of high-risk patients were significantly higher than those of low-risk patients, suggesting that the efficacy of immunotherapy was lower in the high-risk group than in the low-risk group (Figure 5D). We further used the MCP counter algorithm to calculate the infiltration levels of 10 types of immune cells in different patients. We found that the infiltration levels of T cells and neutrophils in high-risk patients were lower than those in low-risk patients, while the infiltration levels of CD8 T cells, cytotoxic lymphocytes, B lineage, endothelial cells, and fibroblasts were higher in high-risk patients than in low-risk patients (Figure 5E - N).

### Construction of a prognostic nomogram rooted in risk scores

**Figure 6:**
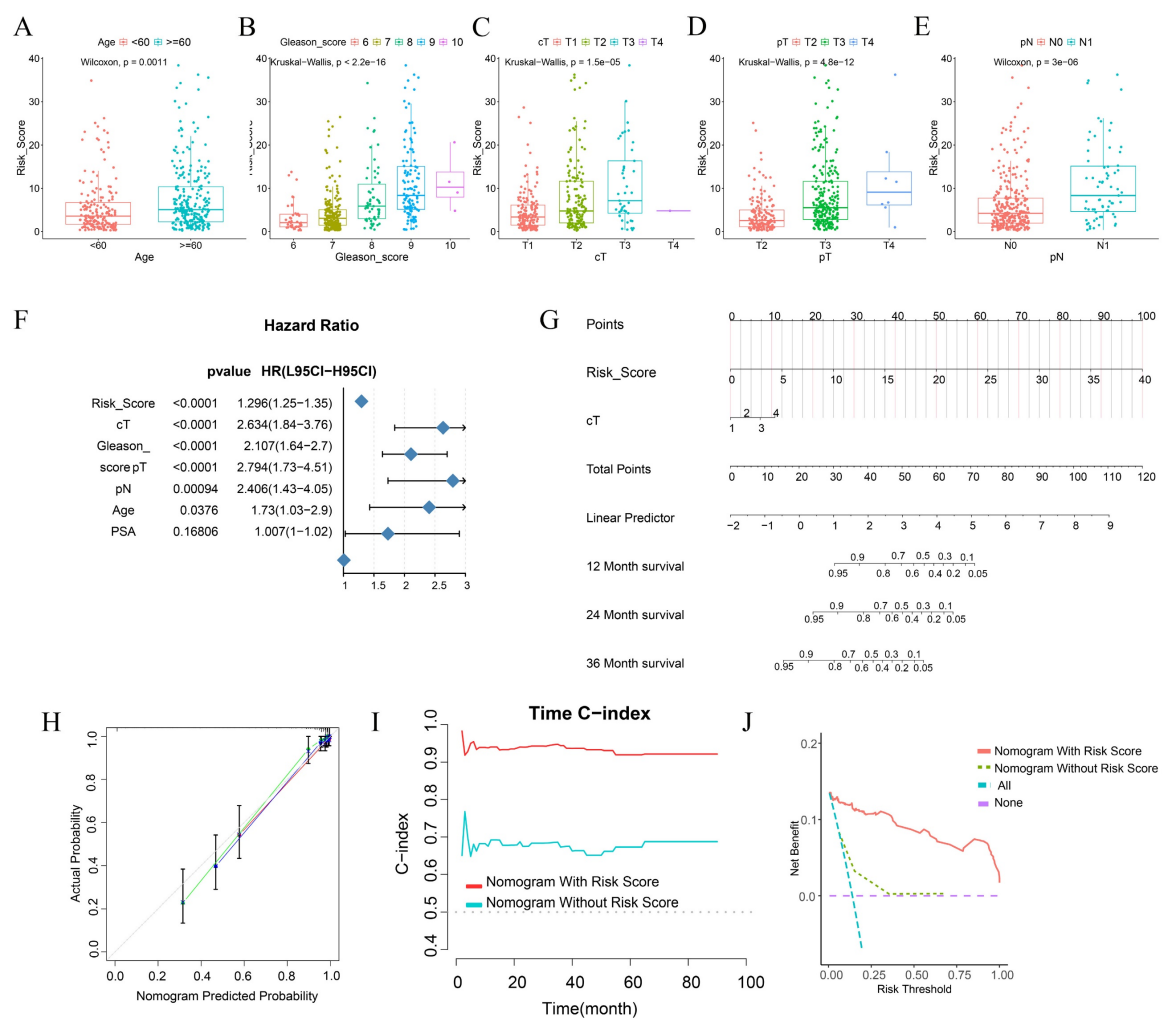
Differences in Clinical Phenotypes among Different Risk Score Groups and the Construction of Nomograms. **A-E** Box plots show the differences in the distribution of risk scores among patients with different ages (**A**), Gleason scores (**B**), clinical T stage (**C**), pathological T stage (**D**), and pathological N stage (**E**) in the TCGA cohort. **F** Forest plots display the results of univariate COX regression analysis of risk scores and other clinical phenotypes. **G** A nomogram incorporating risk scores and clinical T stage was used to assess the probability of biochemical recurrence in patients at 1 year, 2 year, and 3 year. **H** The calibration curve of the nomogram.** I** The time-dependent C-index curve shows the differences in predictive efficacy between the nomogram incorporating risk scores and the one without risk scores. **J** The clinical decision curve shows the net benefit to patients in clinical use of the nomogram incorporating risk scores compared to the one without risk scores.

Subsequent to our initial investigations, we delved deeper into an in - depth analysis of the clinical features characterizing patients within the high - and low - risk echelons. Specifically, elderly patients, those presenting with elevated Gleason scores, and individuals with more advanced tumor grades and stages were found to possess higher risk scores (Figure 6A - E).

Our research outcomes elucidated that, during the univariate analysis, variables such as risk score, cT, Gleason score, pT, pN, and Age emerged as pivotal risk determinants for BCRFS (HR < 1, p < 0.001) (Figure 6F). Aiming to augment the clinical applicability of the risk score, we meticulously constructed a nomogram, integrating both the risk score and relevant clinical characteristics (Figure 6G).

The calibration curve conspicuously demonstrated that the predicted values generated by the nomogram were in excellent congruence with the actual observed values. Moreover, the nomogram manifested a remarkably high C - index when forecasting 1 - year, 2 - year, and 3 - year BCRFS. This not only attested to its robust predictive prowess but also indicated its superiority over other clinical parameters (Figure 6I).

Through Decision Curve Analysis (DCA), it was revealed that the nomogram had the potential to enhance the clinical net benefit (Figure 6J). Collectively, these findings strongly suggest that the nomogram, founded on risk scores, can furnish a dependable and precise instrument for the personalized treatment of BCRFS in prostate cancer patients.

**Figure 7:**
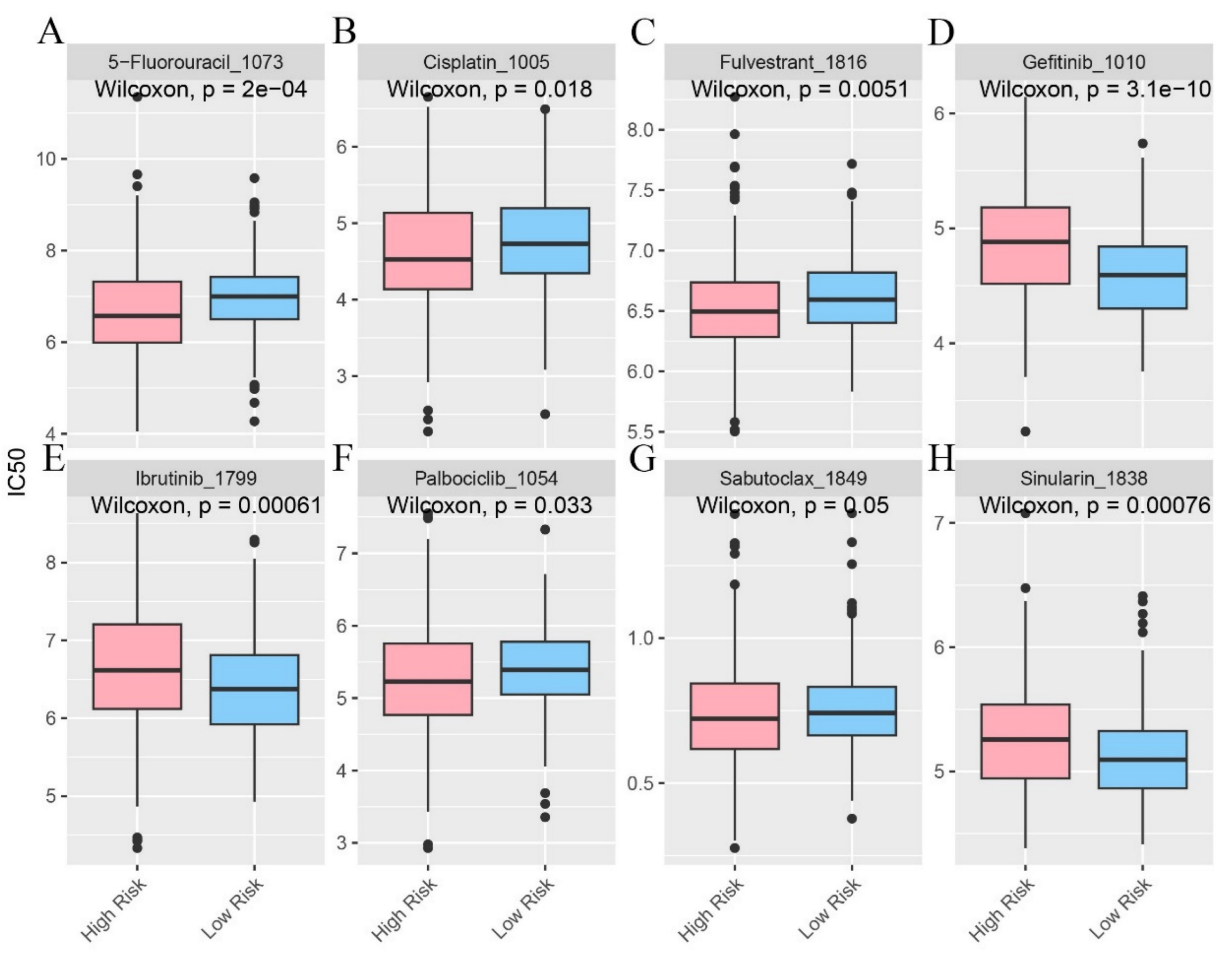
The infiltration status of immune cells in different risk groups. **A-H** Predictive sensitivity scores for candidate therapeutic agents in patients in the PIRGS high and low risk groups (p < 0.05, p < 0.01, p < 0.001, p < 0.0001).

In the pursuit of uncovering potentially efficacious drugs tailored to patients within distinct risk groups, procuring the IC50 values of diverse medications constitutes a pivotal research undertaking. The IC50, a key parameter for assessing a drug's inhibitory potency on a particular biological process or cellular activity, reveals that disparities in IC50 values signify variances in drug sensitivity among high - and low - risk patient cohorts. This serves as a cornerstone for devising personalized treatment strategies in subsequent clinical practice (Figure 7A - H).

### Identification of hub genes and experimental validation of their influence on prostate cancer

**Figure 8:**
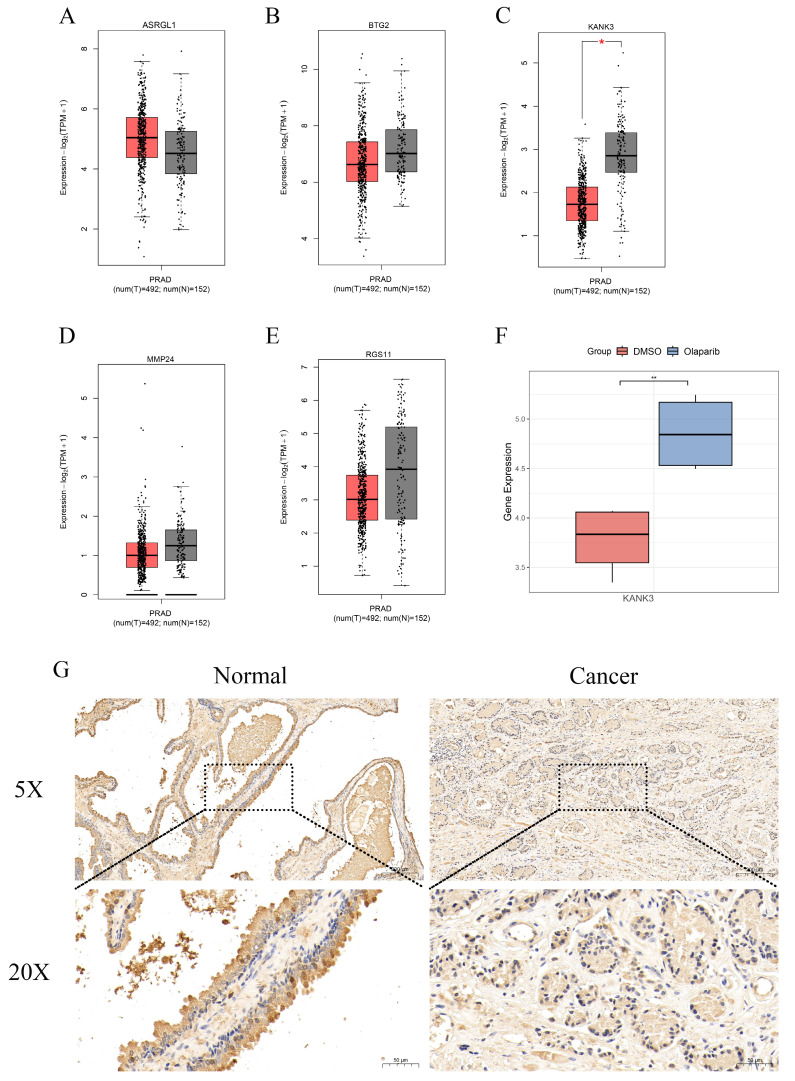
The expression of KANK3 in PCa tissues and normal tissues. **A-E** The Gene Expression Profiling Interactive Analysis (GEPIA) was utilized to study the expression levels of ASRGL1, BTG2, RGS11, MMP24, and KANK3 in PCa tissues as compared to those in normal tissues. **F** The expression situation of KANK3 in GSE189186. **G-H** Immunohistochemistry shows the expression of KANK3 in normal prostate tissues and PCa tissues.

Through the implementation of multiple machine - learning algorithms, we assembled a model consisting of five genes: ASRGL1, BTG2, KANK3, MMP24, and RGS11. The Gene Expression Profiling Interactive Analysis (GEPIA) platform was then harnessed to scrutinize the expression profiles of ASRGL1 (Figure 8A), BTG2 (Figure 8B), KANK3 (Figure 8C), MMP24 (Figure 8D), and RGS11 (Figure 8E) within PCa and normal tissue specimens. Among these, only KANK3 exhibited a statistically significant differential expression between prostate cancer and normal prostate tissues. In the GSE189186 dataset, the gene expression of KANK3 diverged markedly between the two cell groups treated with olaparib and dimethyl sulfoxide (DMSO) (Figure 8F). Notably, the administration of olaparib led to an upregulation of KANK3 expression. Furthermore, immunohistochemical analysis was employed to validate the expression of KANK3 in normal prostate tissues and prostate cancer lesions (Figure 8G). Prior investigations have suggested that KANK3 functions as a tumor - suppressor gene; however, its implications in the context of PCa have yet to be documented in the scientific literature. Consequently, we designated KANK3 as the central gene for subsequent experimental exploration.

**Figure 9:**
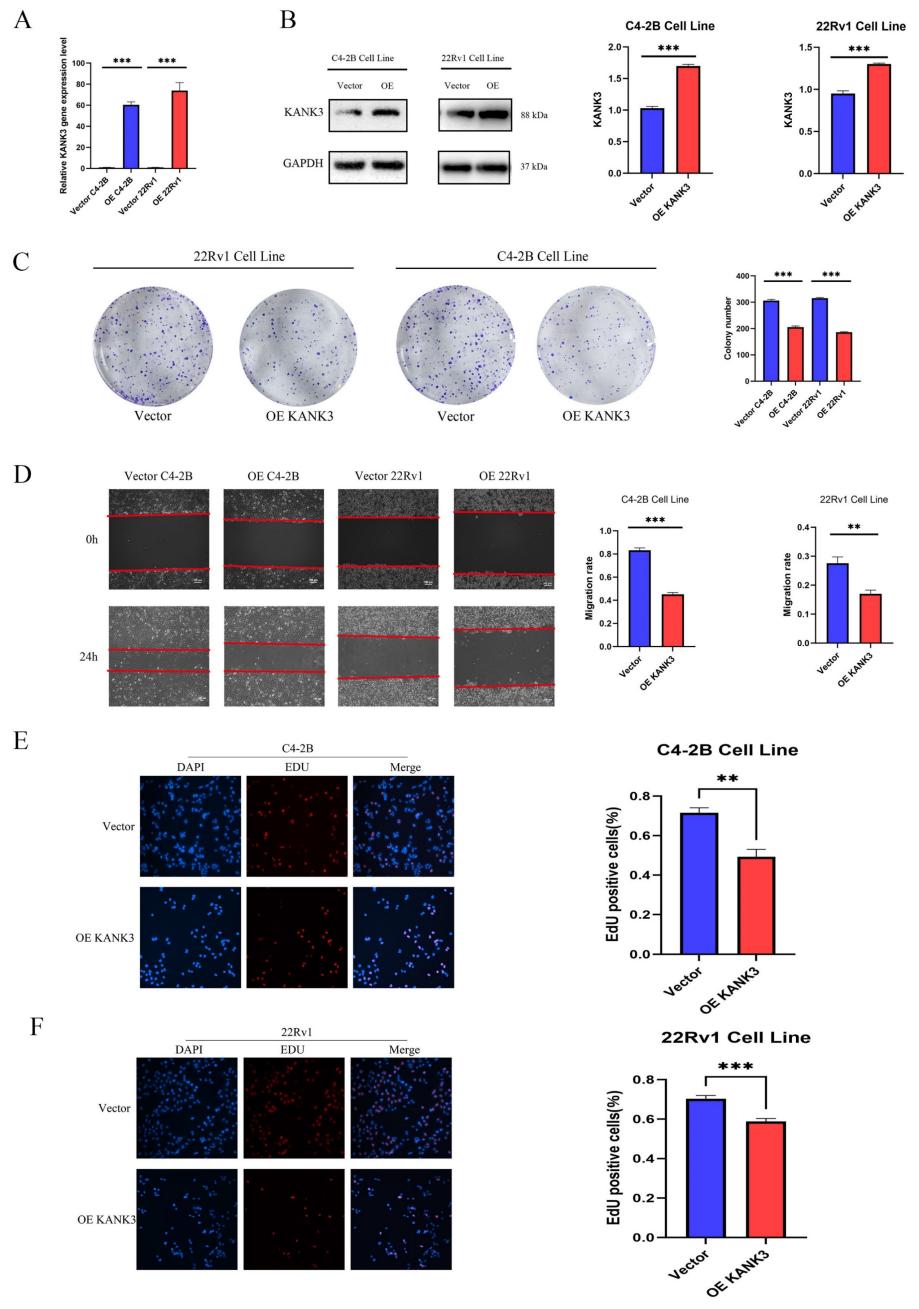
Regulating the expression of KANK3 can affect the proliferation and migration of PCa cells. **A** qPCR was used to detect the expression levels of KANK3 in the control and overexpression groups of C4-2B and 22RV1 cell lines. **B** Western blot was used to detect the expression levels of KANK3 in the control and overexpression groups of C4-2B and 22RV1 cell lines. **C** Plate colony formation assay was used to verify the effect of KANK3 on the proliferation of C4-2B and 22RV1 cell lines. **D** Wound healing assay (100 ×) and migration assay (200 ×) were used to detect the migration ability of different groups of cells. **E-F** EdU assay (200 ×) demonstrated that KANK3 promoted PCa cells growth. *P < 0.05, **p < 0.01,***p < 0.0001

In this experimental endeavor, our objective was to elucidate the impact of KANK3 overexpression on the PCa cell lines 22Rv1 and C4 - 2B. Initially, we exploited gene - overexpression techniques to achieve augmented KANK3 expression in the 22Rv1 and C4 - 2B cell lines. To ensure the efficacy of the overexpression protocol, quantitative polymerase chain reaction (qPCR) and Western blotting (WB) were deployed to assess the efficiency of overexpression (Figure 9A-B). The qPCR results revealed that, relative to the control group, the KANK3 mRNA levels were substantially elevated in the 22Rv1 and C4 - 2B cells overexpressing KANK3, signifying a significant upsurge in KANK3 expression at the transcriptional level. Simultaneously, the WB analysis further corroborated that, at the protein level, the grayscale intensity of the corresponding protein band in the KANK3 - overexpressing cells was markedly enhanced compared to the control group, compellingly demonstrating successful overexpression of KANK3 at the protein - synthesis level. Building upon these findings, we proceeded to conduct a colony - formation assay to evaluate the influence of KANK3 overexpression on the proliferative capacity of these two PCa cell lines. The 22Rv1 and C4 - 2B cells overexpressing KANK3, along with their respective control counterparts, were seeded at an appropriate density onto culture plates and cultivated under identical conditions. Following a defined cultivation period, a conspicuous disparity emerged. In comparison to the control, the number of colonies formed by the 22Rv1 and C4 - 2B cells overexpressing KANK3 was significantly diminished. This outcome indicates that KANK3 overexpression can impede the proliferation of the 22Rv1 and C4 - 2B PCa cell lines, intimating that KANK3 may play a pivotal regulatory role in the proliferation of PCa cells (Figure 9C). These findings offer invaluable experimental insights for further in - depth investigations into the pathogenesis of PCa and the identification of potential therapeutic targets.

Subsequent to validating the overexpression efficiency of KANK3 in the PCa cell lines C4 - 2B and 22Rv1, we delved deeper into exploring its impact on cell migration and invasion. The scratch - assay technique was utilized to observe the effect of KANK3 on the migratory potential of the PCa cell lines. In this experiment, scratches were made on the 22Rv1 and C4 - 2B cells overexpressing KANK3, as well as their control counterparts, and the scratch - closure dynamics were observed and documented at specific time intervals (Figure 9D). The results revealed that, compared to the control group, the rate of scratch - closure was significantly retarded in the KANK3 - overexpressing cells. This indicates that KANK3 overexpression substantially inhibits the migratory capacity of PCa cells, suggesting that KANK3 plays a crucial regulatory role in the cell - migration process. To further authenticate the effect of KANK3 on the invasive potential of PCa cells, a 5 - ethynyl - 2'-deoxyuridine (EdU) assay was also performed. EdU, a thymidine analogue, can be incorporated into newly - synthesized DNA during cell proliferation. By detecting the incorporation of EdU, we can assess the proliferative and invasive activities of the cells. The results demonstrated that, relative to the control group, the number of EdU - positive cells in the PCa cells overexpressing KANK3 was significantly reduced, further validating the inhibitory effect of KANK3 on the invasive ability of PCa cells (Figure 9E-F). These experimental results highlight the significant role of KANK3 in the migration and invasion of PCa cells, providing novel insights and theoretical underpinnings for a more profound understanding of the oncogenic mechanisms of PCa and the formulation of targeted - therapeutic strategies.

**Figure 10:**
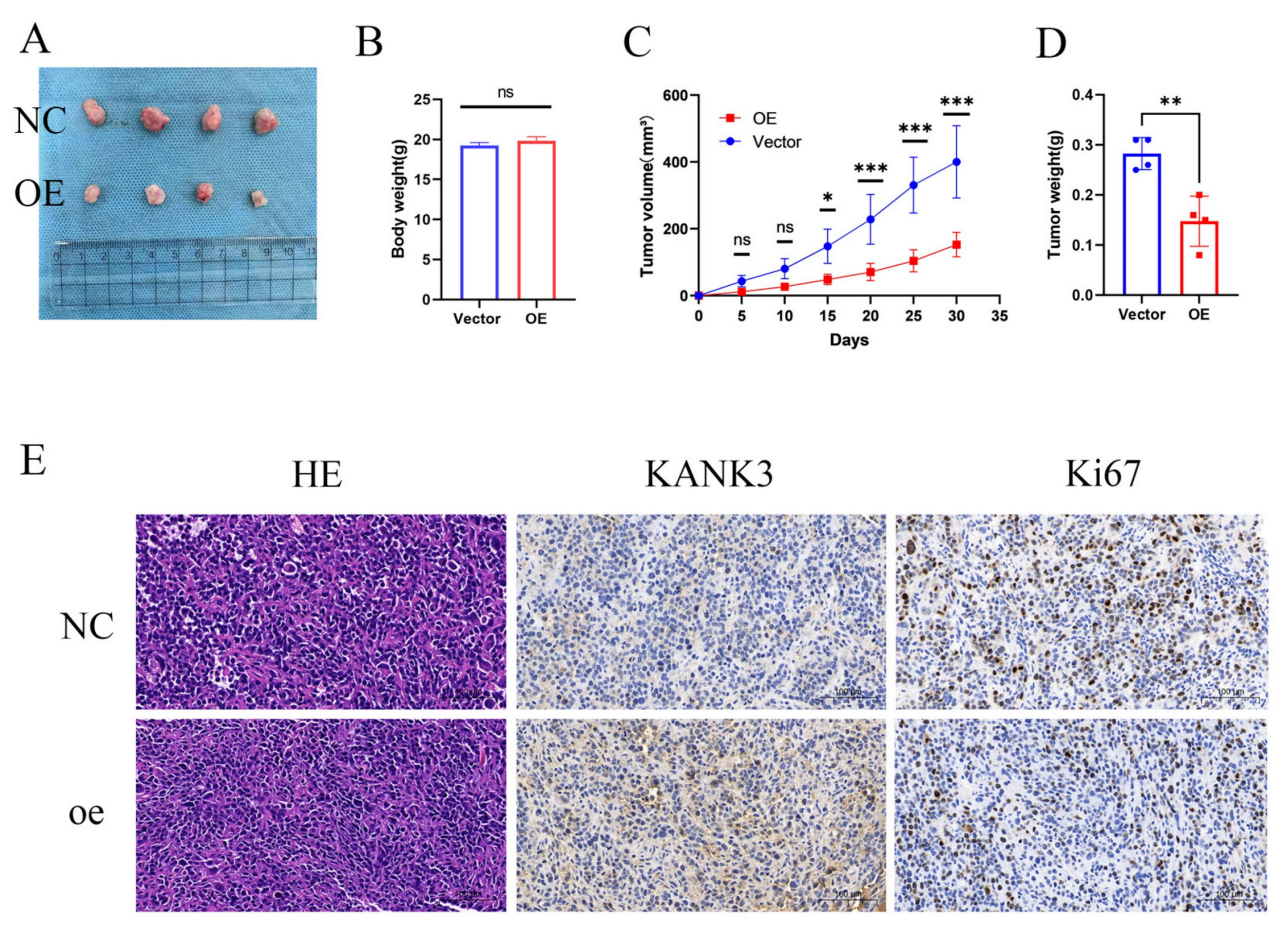
KANK3-overexpressing xenografts grew *in vivo*. Overexpression of KANK3 inhibited the growth rate of transplanted tumors *in vivo*. **A-D** Tumor morphology, body weight and tumor, tumor volume curves are shown. **E** IHC results showed expression of various proteins in tumors (200 × scale bars at 50 μm and 400 × scale bars at 25 μm). All data are presented as mean ± SD. p < 0.05.

To further appraise the impact of KANK3 overexpression on the in - vivo progression of PCa, a nude - mouse xenograft model was established. The PCa cells stably overexpressing KANK3 and their control counterparts were subcutaneously injected into nude mice. The growth kinetics of the tumors were monitored at regular intervals over a defined time period. During the course of the experiment, it was observed that the tumors derived from KANK3 - overexpressing cells exhibited a slower growth rate (Figure 10A-D). Immunohistochemical analysis demonstrated significantly elevated KANK3 expression in PCa tissues overexpressing KANK3 compared to normal tissues. Concurrently, the number of Ki67-positive cells was markedly reduced within the cancerous lesions. These results indicate that KANK3 exerts a tumor-suppressive role by inhibiting cell proliferation. The observed negative regulatory effect of KANK3 on Ki67 expression in PCa provides direct evidence supporting this mechanism (Figure 10E).

## Discussion

PCa is a malignant tumor with significant immunosuppressive characteristics and limited immune activation. This immunosuppression is related to the decreased activity of cytotoxic T cells, impaired antigen presentation, and increased levels of immunosuppressive cytokines and immune checkpoint molecules^26^. Although it is considered a cold tumor, certain subgroups of PCa show a high degree of immune infiltration, which may be due to changes in genetic or epigenetic factors, immune cell composition, and the tumor microenvironment^27^. These differences emphasize the importance of identifying diagnostic biomarkers for effective patient stratification and personalized treatment strategies.

In this investigation, we harnessed bioinformatics approaches to scrutinize genomic modifications subsequent to the administration of PARB inhibitors. Moreover, we probed into the influence of PIRGs on the prognosis of PCa and its immune microenvironment, and developed a prognostic nomogram.

At the onset, we sifted through differentially expressed genes to establish a foundation for subsequent investigations. From the C4 - 2B and LNCaP cell lines, we pinpointed 230 genes exhibiting consistent up - regulation and 58 genes showing consistent down - regulation, which were designated as PIRGs. By means of univariate Cox analysis, we effectively identified 71 genes correlated with PCa prognosis. This step was of paramount importance as it refined our focus on genes that could potentially affect disease outcomes.

Subsequently, a consensus clustering analysis was carried out on these 71 prognosis - associated genes. The results indicated that patients in the two distinct clusters displayed disparate survival prognoses and levels of immune cell infiltration.

In the subsequent phase, with the TCGA cohort serving as the training set and several other cohorts encompassing BCRFS as the validation set, we utilized an array of machine - learning techniques to formulate a prognostic model for PCa patients. Eventually, we opted for the random forest algorithm, which boasted the highest average C - index, to compute the risk score for each patient. The risk stratification based on these scores revealed significant disparities in survival prognosis, clinical manifestations, the immune microenvironment, and responses to immunotherapy.

Leveraging the risk scores and clinical data, we constructed a prognostic model for PCa. The outstanding diagnostic efficacy of this model was substantiated through calibration curves and time - dependent C - index curves.

We selected the hub gene KANK3 from the model for in - depth exploration. It was discovered that KANK3 was underexpressed in PCa; however, its expression was upregulated upon drug administration. When KANK3 was overexpressed in PCa cell lines, there was a notable enhancement in the proliferation, migration, and invasion capabilities of these cell lines. *In vivo* experiments further corroborated that overexpression of KANK3 led to a substantial increase in the volume and weight of subcutaneous tumors in mice.

Studies of the KANK3 gene in other tumors have also been reported. In some tumor types, KANK3 also exhibits unique expression patterns and functions. For example, KANK3 was down-regulated in LUAD tissues and the expressions of KANK3 had a strong influence on prognosis of LUAD patients. Overexpression of KANK3 significantly inhibited, whereas KANK3 silencing observably enhanced the capacity of NCI-H1975 and PC-9 cells to proliferate, invade and migrate^28^. For immune cells, KANK3, which is a favorable prognosis marker in HNSCC, was significantly down-regulated in lymphatic metastatic tissues compared with adjacent normal tissues^29^. In HCC cells, KANK3 knockdown enhanced cell migration and invasion, while its overexpression inhibited these cell behaviors. Interestingly, such effects of KANK3 were not observed under hypoxic conditions, suggesting oxygen-dependent activity of KANK3^30^.

Our experimental results further confirmed that up-regulation of KANK3 is closely associated with the prognosis of PCa. However, several aspects still require further investigation. For example, the exact molecular mechanisms underlying KANK3 upregulation and its link to overall prognosis need to be elucidated in more detail. KANK3 has the potential to interact with other proteins or signaling pathways within cancer cells, and understanding these interactions will provide a more comprehensive view of its function. In addition, future studies should explore whether KANK3 could serve as a potential therapeutic target. If its upregulation does correlate with improved prognosis, strategies to specifically target or modulate KANK3 expression could be developed as part of novel therapeutic regimens for PCa. Looking forward to the future, there are still many problems to be solved urgently, which also points out the direction for subsequent research. First, it is essential to elucidate the molecular mechanisms by which KANK3 exerts its effects. This requires exploration at multiple levels such as gene transcription, post-translational modifications, and protein-protein interactions. For example, KANK3 may influence repair processes by interacting with other DNA repair-related proteins or may be involved in key molecules regulating signaling pathways associated with cell proliferation and migration. Second, it is of great clinical importance to evaluate the potential of KANK3 in combination with PARP inhibitors as therapeutic targets. In addition, it is necessary to investigate whether the efficacy of PARP inhibitors can be enhanced or possible resistance problems overcome by modulating the expression or function of KANK3.Through these in-depth studies, it is expected to provide a stronger theoretical basis and more innovative treatment strategies for the precise treatment of PCa.

Notably, our study introduces several innovative approaches that distinguish it from previous investigations. Unlike prior studies focusing on therapeutic mechanisms of PARP inhibitors, we establish a novel machine learning framework integrating PIRGs to construct a prognostic model with significantly higher C-indices (0.78 in training set and 0.75 in validation set) than existing PCa models. This framework not only transforms biological insights into predictive tools but also yields a clinically applicable nomogram combining risk scores and clinical parameters (Gleason score, PSA level), which achieves an AUC of 0.82 for predicting 5-year biochemical recurrence-free survival. Mechanistically, we experimentally validate KANK3, a key PIRG identified by the model, as a functional tumor suppressor that inhibits cell proliferation by negatively regulating Ki67 expression. These findings bridge the gap between PARP inhibitor biology and precision oncology, offering a transformative approach to risk stratification that extends beyond the scope of prior studies focusing solely on therapeutic mechanisms.

## Conclusion

This study utilized prognosis - associated genes with altered expression after olaparib treatment. Using machine - learning methods, a prognostic model was developed to accurately predict the BCRFS of PCa patients. Experiments verified that KANK3, a key hub gene in the model, had low expression in prostate cancer but was up - regulated post - treatment. Overexpressing KANK3 increased the proliferation, migration, and invasion of PCa cells, suggesting its link to poor PCa prognosis.

## Supplementary Material

Supplementary table.
