# Supplementary material for: Identification and validation of the PARP inhibitor-related gene KANK3 for predicting prognosis and immunotherapeutic response in prostate cancer
Source: J Cancer. 2025 Sep 3;16(13):3942–59. doi: 10.7150/jca.113546 (PMC12491201; doi:10.7150/jca.113546)
Supplement: Supplementary file 1 — Supplementary table. [file jcav16p3942s1.pdf]

Supplementary Table 1: Baseline Data Table of the Dataset Used in This Study

Characteristic	Chinese_PRAD<br>N = 116[1]	DKFZ2018<br>N = 268[1]	GSE70768<br>N = 125[1]	GSE70769<br>N = 94[1]	TCGA<br>N = 484[1]
Age (years)	69 ± 6	53 ± 10	62 ± 8	-	61 ± 7
PSA (ng/mL)	44 ± 124	25 ± 59	18 ± 42	11 ± 13	2 ± 16
Clinical T stage	-	-	-	-	-
T1	35 (31%)	1 (0.4%)	62 (56%)	41 (46%)	176 (44%)
T2	58 (51%)	172 (64%)	33 (30%)	39 (44%)	171 (43%)
T3	21 (18%)	82 (31%)	16 (14%)	9 (10%)	50 (13%)
T4	0 (0%)	13 (4.9%)	0 (0%)	0 (0%)	2 (0.5%)
Clinical N stage	-	-	-	-	-
N0	104 (91%)	-	-	-	-
N1	10 (8.8%)	-	-	-	-
Clinical M stage	-	-	-	-	-
M0	105 (94%)	-	-	-	-
M1	7 (6.3%)	-	-	-	-
Pathological T stage	-	-	-	-	-
T2	59 (51%)	-	33 (30%)	48 (53%)	187 (39%)
T3	52 (45%)	-	75 (68%)	42 (47%)	280 (59%)
T4	5 (4.3%)	-	2 (1.8%)	0 (0%)	10 (2.1%)
Pathological N stage	-	-	-	-	-

Supplementary Table 1: Baseline Data Table of the Dataset Used in This Study

**Characteristic**	**Chinese_PRAD** N = 116[1]	**DKFZ2018** N = 268[1]	**GSE70768** N = 125[1]	**GSE70769** N = 94[1]	**TCGA** N = 484[1]
N0	83 (86%)	-	-	-	339 (82%)
N1	14 (14%)	-	-	-	76 (18%)
Pathological M stage	-	-	-	-	-
M0	55 (80%)	-	-	-	-
M1	14 (20%)	-	-	-	-
Gleason Score	-	-	-	-	-
5	0 (0%)	0 (0%)	0 (0%)	2 (2.2%)	0 (0%)
6	10 (8.6%)	38 (14%)	17 (14%)	18 (20%)	45 (9.3%)
7	53 (46%)	179 (67%)	87 (71%)	56 (61%)	242 (50%)
8	17 (15%)	5 (1.9%)	9 (7.4%)	5 (5.4%)	63 (13%)
9	36 (31%)	43 (16%)	7 (5.7%)	9 (9.8%)	130 (27%)
10	0 (0%)	3 (1.1%)	2 (1.6%)	2 (2.2%)	4 (0.8%)

[1]Mean ± SD; n (%)
